# Exploring perceptions towards health and child nutrition: A qualitative study among tribal mothers in Southern Karnataka

**DOI:** 10.1371/journal.pone.0351319

**Published:** 2026-06-23

**Authors:** Sowmya Pujari, Ranjitha S. Shetty, Suneel C. Mundkur, Elsa Sanatombi Devi, Amol Dongre, B. Unnikrishnan, Sreedharan Nair

**Affiliations:** 1 Department of Pharmacy Practice, Manipal College of Pharmaceutical Sciences, Manipal Academy of Higher Education, Manipal, India; 2 Department of Community Medicine, Kasturba Medical College, Manipal Academy of Higher Education, Manipal, India; 3 Department of Paediatrics, Kasturba Medical College, Manipal Academy of Higher Education, Manipal, India; 4 Department of Medical Surgical Nursing, Manipal College of Nursing, Manipal Academy of Higher Education, Manipal, India; 5 Department of Community Medicine, Sri Manakula Vinayagar Medical College, Puducherry, India; 6 Department of Community Medicine, Kasturba Medical College, Mangalore, Manipal Academy of Higher Education, Manipal, India; Kasturba Medical College Manipal, INDIA

## Abstract

**Background:**

Malnutrition accounts for nearly one-third of child deaths globally and continues to be a major concern in India. Despite economic progress, undernutrition remains prevalent, with one-third of children underweight and over two-thirds anemic. Within India, marginalized groups such as the Koraga tribe face greater risks due to poverty, limited healthcare access and cultural barriers that compound child health challenges. Understanding maternal perceptions is crucial to effectively address these challenges.

**Methods:**

In-depth interviews were conducted with Koraga tribal mothers of children aged 5–10 years. Participants were selected using criterion-based purposive sampling to ensure representation across different age groups and household contexts. Interviews were audio-recorded in local languages (Tulu and Kannada), transcribed verbatim, translated into English and analysed inductively using thematic analysis with NVivo software (version 14).

**Results:**

Twenty Koraga tribal mothers were interviewed between October 2023- March 2024. Thematic analysis revealed five major themes: evolving perceptions of health and wellbeing; nutrition beliefs and practices; hygiene and health promotion; traditional healing with modern care and barriers and community solutions. These findings highlighted key challenges such as limited healthcare access, poverty and educational constraints, while also capturing community-driven strategies including reliance on health workers and government food-schemes.

**Conclusion:**

Maternal perceptions, shaped by cultural norms and socioeconomic constraints, play a critical role in influencing health and child nutrition in the Koraga community. The findings highlight the need for policy measures that integrate culturally informed nutrition education with strengthened frontline health services, alongside community-based programs involving women’s self-help groups to improve health outcomes in marginalised tribal populations.

## 1. Introduction

Nutrition plays a key role in shaping an individual’s overall health and wellbeing [1]. While the physical effects of undernutrition like stunted growth, poor immune system and impaired cognitive development are widely recognised [2], there remains a critical need to understand the social and cultural factors that influence nutritional outcomes, especially in marginalized communities [3]. In India, these vulnerabilities are particularly pronounced among tribal populations, who face persistent socio-economic disadvantages that heighten the risk of undernutrition in children [4].

India is home to approximately 104 million tribal people, accounting for 8.6% of the country’s total population [5]. Among the 705 Scheduled Tribes (STs), 75 groups have been classified as Particularly Vulnerable Tribal Groups (PVTGs) by Government of India, due to the distinct social-economic disadvantages they experience. One such group is the Koraga community in Karnataka, where undernutrition, restricted access to healthcare services, and limited awareness of hygiene practices such as sanitation and oral hygiene remain ongoing challenges [6–8].

Most national and state-level nutrition data, including the National Family Health Surveys (NFHS) [9], focus primarily on children below 5 years of age. However, the 5–10 year age group represents a critical but often overlooked phase of middle childhood. This stage is characterised by increased nutritional requirements, rapid physical growth, and school enrolment, yet receives limited programmatic and research attention. Inadequate dietary intake at this stage leads to retarded growth, cognitive impairment and poor academic performance [10,11].

Accordingly, the study focused on children aged 5–10 years, complementing existing evidence by capturing how nutritional vulnerabilities may persist or evolve beyond early childhood, particularly in marginalized communities where inadequacies in dietary intake and health support may continue into school-going years.

A previous study conducted by Pujari et al. reported a high burden of malnutrition among Koraga children aged 5–10 years. Stunting was observed in 16.7% of children, while 35.7% were underweight and 84.6% were anaemic [12]. These rates are considerably higher than the Karnataka state averages among children aged below 5 years (35.4% stunting, 32.9% underweight and 65.5% anaemic) [9]. Although there is no specific state-level data for children aged 5–10 years, the comparison highlights that Koraga children experience disproportionately higher levels of malnutrition and anaemia, suggesting that vulnerabilities persist beyond early childhood. In addition to poor nutrition, structural and behavioural risk factors such as alcohol and tobacco use, social marginalization and geographic isolation worsen health outcomes in this community [8].

Although quantitative studies have reported the prevalence of malnutrition in Koraga children, for instance, an anthropometric study conducted by Pujar et al. documented that children suffered from different grades of malnutrition [13], there is limited information on caregiving practices and maternal perspectives. Maternal perceptions have a significant impact on health-decision making at the household-level, affecting aspects such as dietary choices, hygiene behaviours and healthcare-seeking patterns [14]. However, there is still a lack of research focusing on personal and cultural viewpoints that underlie these practices in the Koraga community. Understanding these perceptions is essential for developing culturally appropriate and community-specific interventions. Relying solely on quantitative data may often overlook the underlying socio-cultural beliefs and barriers that shape how health and nutrition are perceived and managed.

In this context, the present study aimed to explore the perceptions of Koraga tribal mothers regarding health and nutritional well-being of children aged 5–10 years.

## 2. Methodology

### 2.1 Study design and population

A qualitative ethnographic study, grounded in the constructivist paradigm, was carried out among mothers of children aged 5–10 years from the Koraga tribal community. This paradigm was particularly suitable for our study as it allowed us to capture the beliefs and perceptions that Koraga mothers attributed to child health and nutrition, rather than imposing predefined categories [15].

The study was conducted among the Koraga community residing in rural villages of the Udupi district, coastal Karnataka, India. Udupi district is located in the southern coastal region of Karnataka and has a population of 1,177,361 [16]. The Koragas typically live in small settlements called colonies located on the fringes of villages, with limited access to health and nutrition services [4,17,18]. Mothers of children aged 5–10 years were purposively selected because this is a critical period of middle childhood, when nutritional demands increase due to rapid growth and school entry [10]. Mothers in their third trimester of pregnancy or within two-three months postpartum were excluded from the study. The study was reported in accordance with the Consolidated Criteria for Reporting Qualitative Research (COREQ) with reporting summarized in S1 File [19].

### 2.2 Ethical considerations

Approval for the study was obtained from the Kasturba Medical College and Kasturba Hospital Institutional Ethics Committee, Manipal (IEC-381/2022 [03-04-2023]). A detailed explanation of the study was provided in the local language, and written informed consent was obtained from the mothers before their enrolment. The research was conducted in accordance with the Declaration of Helsinki. In addition, administrative approvals were obtained from the Department of Health and Family Welfare, Government of Karnataka; The Integrated Tribal Development Project (ITDP), Udupi District; and the District Health Officer, Udupi District.

### 2.3 Sample size

During the course of data collection, sufficient richness of information was achieved by the 18^th^ interview, as no new insights emerged. Two additional interviews were conducted to ensure completeness and confirm that key perspectives had been captured. Sample adequacy was assessed at two levels: code saturation, when no new codes were generated during the iterative coding process, and meaning saturation, when further data collection did not yield additional insights. This approach aligns with the recommendations of Hennink MM, Kaiser BN and Marconi VC, who identified these two as key indicators for determining sample adequacy in qualitative research [20].

### 2.4 Sampling technique

A criterion-based purposive sampling technique was employed to recruit eligible participants who could provide relevant and diverse perspectives on health and child nutrition within the Koraga tribal community [21]. Mothers were approached with the help of ASHAs and tribal leaders, who facilitated initial contact and identified households with children aged 5–10 years. Participation was guided by mother’s willingness and ability to share their experiences and perspectives during interviews, ensuring inclusion of diverse viewpoints within the community. Efforts were made to include mothers across varying ages, educational backgrounds and household contexts to capture a broad range of perceptions and minimize selection bias.

### 2.5 Role of research team

The research team comprised investigators from multidisciplinary backgrounds, including community medicine, paediatrics, nursing, and pharmacy practice, with prior experience in qualitative research and public health. The team included women researchers, a physician scientist (RS), a nursing researcher (ES), and a research scholar (SP). ES, RS, and AD have published studies using qualitative research methods. None of the researchers had any prior relationship with the participants.

The lead researcher (SP), a female PhD scholar trained in qualitative research, conducted all interviews and was responsible for transcription and translation of the data. She was fluent in the local languages (Tulu and Kannada) and spent approximately five months embedded within the Koraga community, facilitating rapport and contextual understanding. Her positionality included both insider (linguistic and cultural familiarity) and outsider (academic affiliation) perspectives, which may have influenced participant responses and interpretation. Being a female researcher interviewing mothers likely enabled open communication. The transcripts were cross-checked for accuracy by other members of the research team.

To minimise bias, reflexive practices were adopted, including maintaining field notes and independent coding. Any discrepancies were resolved through discussion and consensus among researchers. This iterative and collaborative approach ensured that interpretations remained grounded in participant narratives rather than researcher assumptions.

### 2.6 Designing and conducting semi structured interview

#### Step 1: Interview guide development.

A semi-structured interview guide was developed through a literature search and discussions with experts from both academic and healthcare fields. It included questions regarding health, nutrition, eating patterns, lifestyle practices, cultural practices, awareness of malnutrition, diet management, and government programmes and policies along with basic demographic details. Examples of questions included: *“What does good health mean to you?”, “Describe the strategies or meal plans that you follow to make sure children receive a variety of nutrients?”* and *“How do you handle challenges related to picky eating or dietary preferences with your* *child?”* Additionally, open-ended questions were added to the interview guide to collect the detailed perceptions of the participants.

#### Step 2: Interview guide validation.

The guide was validated using the interview protocol refinement method [22], which involved four phases:

1. Confirming the alignment of interview questions and the research questions: Interview and research questions were mapped into a matrix across domains such as background, health, nutrition, lifestyle, hygiene, culture and awareness.
2. Building an inquiry-based conversation: This was achieved by including introductory, transition, key and closing questions.
3. Collecting feedback on the interview guide: Six faculty members from Departments of Nursing, Community Medicine, Pharmacy Practice and Public Health reviewed the guide. Their feedback was incorporated and the guide was translated into Tulu and Kannada. The translated version was reviewed by a bilingual expert for accuracy and cultural relevance
4. Piloting the interview guide: The interview guide was piloted among ten participants to assess clarity and ease of understanding. Minor wording changes were made after piloting to improve the clarity; however, no substantial modifications were required.

The complete English version of the semi-structured interview guide used in this study is provided as S2 File.

#### Step 3: Data collection.

Data collection was guided by key principles of ethnographic research, including prolonged engagement, rapport building, participant observation and attention to cultural context. The lead researcher, a PhD student fluent in Tulu and Kannada, spent extended periods in the study settings to facilitate trust, familiarity, open communication with participants. Fieldwork for this qualitative phase lasted for an average of five months (October 2023- March 2024), during which the researcher engaged closely with the mothers to develop trust and gain a deeper understanding of their social and cultural aspects. This prolonged immersion involved both formal interviews and informal interactions, allowing the researcher to observe daily lifestyle practices, build meaningful relationships and be actively involved in local settings. These efforts ensured that the data collected reflected social meanings and everyday practices within the participant’s natural cultural environment. The process was conducted under the guidance of the fourth author, a professor and an experienced qualitative researcher, who provided mentorship to ensure cultural sensitivity and methodological rigor.

Data collection took place across ten Koraga settlements, purposively selected to capture geographical diversity, socio-cultural variation and differing access to health and welfare services. In each settlement, 1–3 mothers of children aged 5–10 years were interviewed through in-depth, semi-structured, face-to-face interviews conducted in person by the lead researcher in the privacy of their homes. Each interview lasted approximately 40–45 minutes. Interviews continued until data saturation was reached by the 18th interview, as no new insights emerged. To confirm that all key perspectives had been captured, two additional interviews were conducted, resulting in a total of 20 interviews. Field notes were taken, and interviews were audio-recorded with informed consent. The pilot interviews were not included in the final analysis.

Data confidentiality was strictly maintained. All recordings and notes were anonymized and stored securely in a password-protected file on an encrypted device. In line with Institutional Ethics Committee guidelines, data were retained for three years before being securely destroyed.

### 2.7 Analysis

Thematic analysis was employed to analyse the qualitative data collected through semi-structured interviews with 20 mothers. The analysis followed a six-step framework outlined by Braun and Clarke (2006), using a transparent and inductive approach in which themes were actively identified, developed, and refined through systematic engagement with the data [23]. The initial coding and theme development were conducted independently by two authors to enhance credibility. Both authors reviewed and discussed the codes and themes iteratively, resolving discrepancies through consensus. This collaborative process ensured transparency and rigour in the identification and interpretation of key patterns across the dataset.

The following steps, consistent with Braun and Clarke’s framework, were applied:

1 Transcription, familiarization with the data and selection of quotations

All interviews were audio-recorded and transcribed verbatim in local languages (Tulu, Kannada) by the first author, who was fluent in both languages. The transcripts captured participant’s words exactly as spoken, including pauses and expressions, to preserve authenticity. To enhance accuracy, the second and third authors cross-checked each transcript against the audio-recordings multiple times. The transcripts were then translated into English, and the translations were reviewed by a bilingual expert to ensure linguistic accuracy. During this process, key quotations were highlighted to reflect diverse viewpoints.

2 Selection of keywords

During the process of familiarization, recurring words and expressions were identified from the transcripts. The terms that represented the participant’s daily practices, cultural beliefs and perceptions were noted as keywords. These keywords formed the basis for developing meaningful codes.

3 Coding

Data coding was conducted systematically using NVivo version 14 software (QSR International Pvt Ltd) [24]. An inductive open coding approach was employed, which allowed codes to be generated from the data without applying a pre-existing coding framework [23]. This approach was chosen to ensure that the analysis remained grounded in participant’s experiences and perspectives. Each code represented a meaningful segment relevant to the research objective. The keywords identified earlier were important in forming and refining the codes. The first and second authors independently coded the transcripts. To ensure consistency, a subset of transcripts was cross-checked by the third author, and discrepancies were discussed until consensus was reached. This iterative process helped refine the codebook and enhance reliability. In total, 337 unique codes were developed during the initial coding phase.

4 Theme development

These 337 codes were then reviewed for conceptual similarities and organized into 17 subthemes, which captured recurring patterns and ideas across participants. Related subthemes were further grouped to form five overarching themes. Thus, the process moved from codes◊ subthemes ◊ themes, ensuring that each level reflected deeper understanding about participant’s experiences. The initial subthemes were drafted by the first author and subsequently reviewed by the fourth author, an experienced qualitative researcher, for conceptual clarity. Final subthemes and themes were developed collaboratively by all authors through iterative discussion, ensuring reflexivity and agreement within the research team.

5 Conceptualization through interpretation of keywords, codes, and themes

Each theme was thoroughly defined and conceptually aligned with the objective of the study. Meanwhile, subthemes were refined through collaborative discussion among all authors to enhance clarity and validity.

6 Thematic integration and Conceptual framework development

The final step focused on integrating the themes and subthemes into a conceptual framework that reflects the maternal perceptions influencing child health and nutrition in the Koraga tribal community. To visually represent this synthesis, a conceptual framework diagram was developed based on the generated themes and adapted from the UNICEF framework for child health and nutrition [25]. While the development of a conceptual framework was not the primary objective, it was generated from the thematic analysis and served to enhance interpretation and clarity.

## 3. Results

### 3.1 Demographic characteristics

A total of 20 mothers aged 28–49 years (mean age 34.50 ± 5.06 years) participated in the interviews. Of these, 11(55%) had completed primary education and 9(45%) had attained higher secondary education, with none having received graduation-level education. Most were homemakers 12(60%), while 5(25%) held government or private jobs, and 3(15%) worked in other sectors. In terms of family structure, over half of the participants 11(55%) belonged to nuclear families, 5(25%) to extended families and 4(20%) to joint families. Across these households, there were 23 children aged 5–10 years, with most families having one or two children in this age group. All participants 20(100%) reported mixed dietary habits, including both vegetarian and non-vegetarian foods, and had access to indoor toilet facilities. A detailed demographic profile for each participant is provided in S3 Table.

### 3.2 Themes and sub-themes

The thematic analysis generated five major themes and 17 subthemes that reflected mother’s perspectives, cultural understandings and caregiving practices. The themes included: evolving perception of health and wellbeing; nutrition beliefs and practices; hygiene and health promotion; traditional healing with modern care and barriers and community solutions.

**Fig 1:**
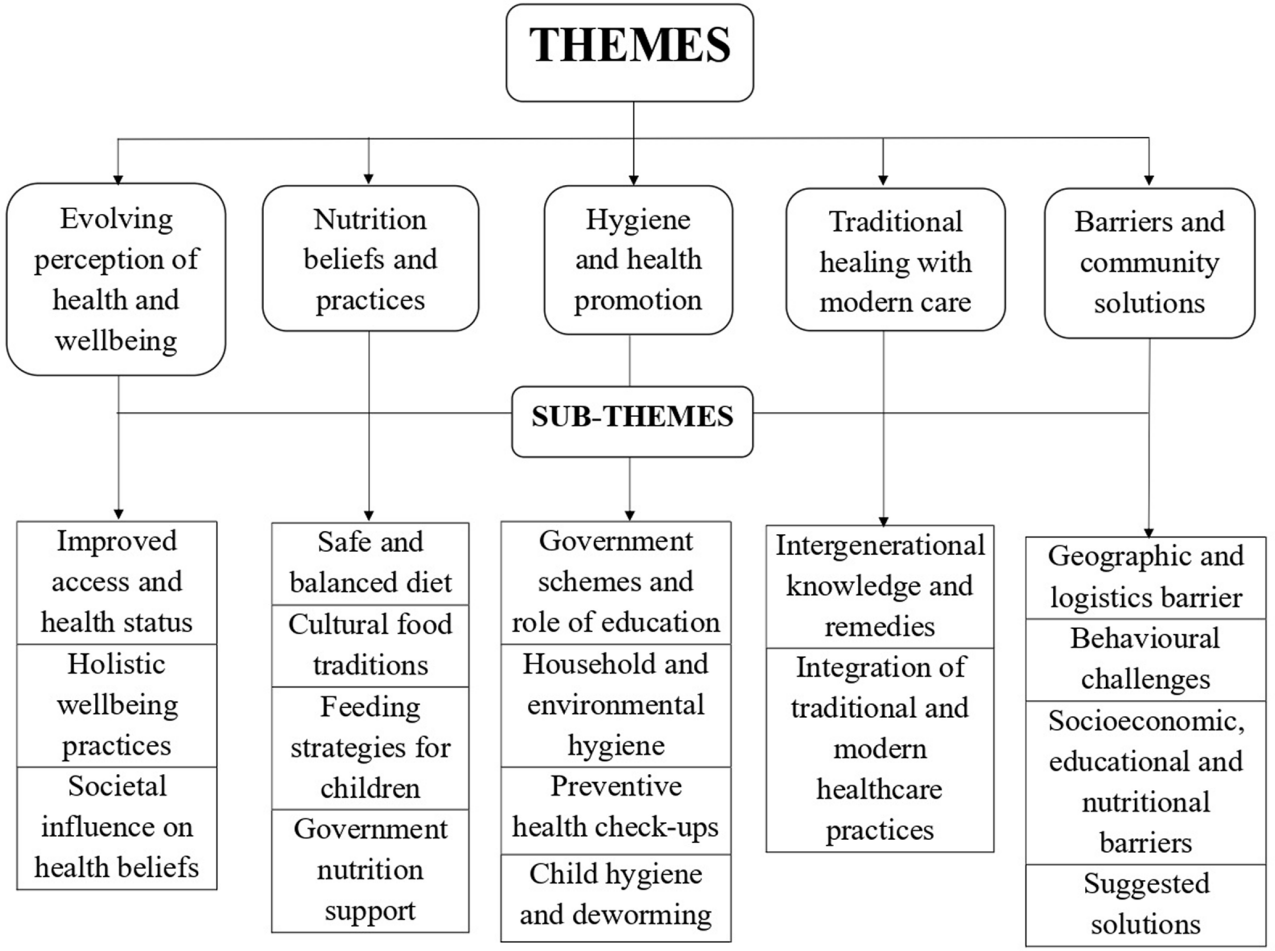
Participant’s perception presented as themes and subthemes (N = 20).

**Fig 2:**
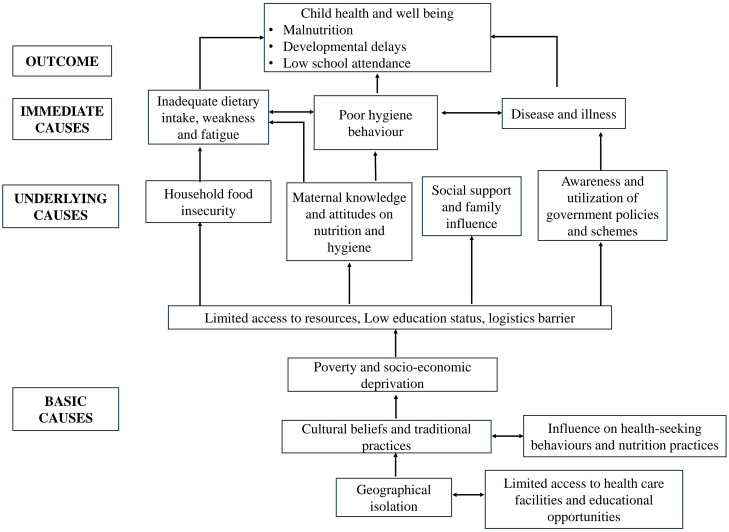
Conceptual framework of maternal perceptions influencing child health and nutritional status among Koraga tribal mothers. Source: Adapted from UNICEF (2020).

An overview of the identified themes and subthemes is presented in Fig 1. To interpret the pathways influencing child health outcomes, we adapted the UNICEF conceptual framework. This framework helped organize the findings under basic, underlying and immediate causes influencing child health and nutritional status in the Koraga tribal community (Fig 2). Illustrative participant quotations are provided in S4 Table using the format: KTP_18_28F = Koraga Tribe Participant 18, aged 28 years, Female.

#### 1. Theme 1: Evolving perception of health and wellbeing.

1.1**Subtheme 1: Improved access and health status**

Mothers generally perceived that the health status of their community had improved compared to the past. They attributed this change to reduced health challenges and the regular visits of ASHA workers, who provided support and linked them to healthcare services. In the past, they recalled facing frequent illness episodes and minimal contact with health workers, which often resulted in worsening conditions before care was sought. Limited awareness about preventive practices was also identified as a challenge. While many difficulties were recalled from earlier times, they emphasized that the situation had improved considerably. One mother shared,

*“Now it is fine, there is no difficulty in health like before. Now, the healthcare centre gets to know about it, it is good for people who have BP and sugar. They come to the house, because of which it is better now.”* [KTP_19_36F]

**1.2 Subtheme 2: Holistic wellbeing practices**

Mothers described health as a state of balance that included cleanliness, nutritious food, adequate sleep and physical activity. They emphasized the need to protect children from infections by being cautious when they were unwell, and by maintaining hygienic practices. Eating food that “cool the body”, drinking water regularly, and avoiding oily foods were highlighted as preventive measures. Yoga, meditation, and daily exercise were also viewed as important for maintaining both physical and mental well-being. One mother explained:

*“We should eat vegetables, fish and meat. Then, we should walk. We must have our food on time. Sleep. We should sleep well and drink plenty of water at regular intervals. That’s all.”* [KTP_11_31F]

Another added, *“We should eat good food. It is good if we exercise and meditate. We should not eat oily food.”* [KTP_13_42F]

**1.3 Subtheme 3: Societal influence on health beliefs**

Mothers perceived that their health beliefs were shaped by a combination of intergenerational teachings, traditional wisdom, and advice from health professionals. Cleanliness and household health practices were learned from parents and grandparents, while nurses, doctors and community meetings reinforced these practices and encouraged healthier behaviours. As one mother explained:

*“Nurses come home, and doctors too visit us. We observe, and they too tell us.”* [KTP_13_42F]

#### 2. Theme 2: Nutrition beliefs and practices.

2.1**Subtheme 1: Safe and balanced diet**

Mothers emphasized that food should be consumed in moderation to maintain balance in the body. Excessive spicy or oily foods were thought to generate “heat”, leading to discomfort and illness. Proper cleaning and safe storage of food were seen as essential to prevent stomach problems. They identified a variety of foods as beneficial for children’s health, including green gram, eggs, milk, bananas, chicken and fish, with eggs and milk often highlighted as important for strength and growth. Traditional staples such as *ganji* (rice porridge) and chilli tamarind paste also remained part of their regular diets.

At the same time, mothers expressed concerns about foods considered harmful, particularly oily foods, potatoes and sweets, which they associated with gastric discomfort, tooth decay, and worm infestations.

One mother explained, *“Green gram, eggs and all we should give. Milk should be given to children after they finish their meal and before they sleep. They eat bananas also. That’s also good for health know. Then, non-vegeterian food items like chicken and fish we should give.”* [KTP_1_ 42F]

Another added, *“Leafy vegetables, then food that cools our body, tender coconut, grapes. If we give all these it is good for their health.”* [KTP_10_38F]

**2.2 Subtheme 2: Cultural food traditions**

Mothers expressed the importance of including traditional dishes in their diet, considering them both nutritious and culturally significant. They described preparing foods such as ‘*gatti*’ (steamed rice dumplings with grated coconut and jaggery filling, wrapped in banana or turmeric leaves), ‘*pathrode*’ (steamed roll made with rice, spices, coconut and locally available *colocasia* leaves), and ‘*dosa*’ (fermented rice and black gram pancakes), often enriched with *colocasia* or moringa leaves. Dishes like ‘*pundi*’ (rice dumplings with coconut and jaggery) and ‘*idli*’, (steamed cakes prepared from fermented batter of rice and black gram) were also prepared for children. As one mother mentioned,

*“We make ‘gatti’ using different leaves like banana leaves and turmeric leaves. Then, we prepare ‘pathrode’, ‘dosa’ made up of ‘kesu’ and drumstick leaves and give it to our children; that’s also good.”* [KTP_1_42F]

**2.3 Subtheme 3: Feeding strategies for children**

Mothers frequently expressed concerns that their children appeared weak, tired easily or preferred unhealthy foods such as sweets, oily snacks and street food. One mother shared:

“*He is little weak. He doesn’t eat properly. Only if we scold him, he eats. He has his food. But he eats more sweets, oily food and street food.”* [KTP_11_31F]

To address these challenges, mothers adopted creative strategies to encourage healthier eating. They blended often disliked but nutritious vegetables into juices with milk, offered alternatives like omelettes instead of boiled eggs, and tried to provide foods that were more acceptable to children. These adaptations were seen as practical ways to improve children’s nutrition while respecting their preferences.

**2.4 Subtheme 4: Government nutrition support**

Mothers recognized the role of government programs in improving their family’s diet. They highlighted the role of ITDP, which provided monthly rations such as pulses, jaggery, eggs, oil and ghee. These supplements were seen as particularly beneficial for children’s nutrition and overall household food security.

As one mother explained, “*We get food, you know-jaggery, and all. For our Koraga community we get monthly nutritious food from -ITDP-. In that, we get chickpeas, toor dal, jaggery, eggs and ghee.”* [KTP_14_42F]

#### 3. Theme 3: Hygiene and health promotion.

**3.1 Subtheme 1: Government schemes and role of education**

Mothers described being informed about government programs that support maternal and child health, particularly the ‘*Thayi Card*’, which provides nutritional and health benefits during pregnancy and early childhood. Awareness of these schemes was often facilitated by ASHA workers, nurses, and community members, who visited households to share information and encourage participation. These interactions were seen as an important source of guidance about available services.

At the same time, mothers highlighted the role of education in shaping health practices. Lessons in schools about cleanliness and personal hygiene, were viewed as important for preventing infections. They also noted that, children were regularly given deworming tablets at school, which increased parental awareness of worm-related health issues. This awareness helped families recognize symptoms such as stomach pain or vomiting and seek timely care when needed. One mother explained, *“[birds chirping] Thayi card. It has been very useful for us. We have been following that.”* [KTP_13_42F]

Another added*, “Yes, education is important. It is very important. If stomach worm increases, children’s stomach starts to pain, they will vomit. All this we understand and get them medicines.”* [KTP_10_38F]

**3.2 Subtheme 2: Household and environmental hygiene**

Mothers stressed the importance of keeping both the home and surroundings clean to prevent illness. They associated stagnant water with the breeding grounds of mosquitoes and the spread of infections. They also emphasised on regular cleaning of kitchens, bathrooms and toilets. Everyday practices such as washing vegetables, changing clothes, trimming nails, and maintaining pet hygiene were described as part of protecting children’s health. Personal routines like brushing teeth, bathing and frequent handwashing were widely encouraged. One mother shared,

*“For dust, it is good to clean. We should not allow dirty water to stay for long time; otherwise, mosquitoes will increase, and they will bite us and cause problems for our health. So, I fear that. If we keep clean, then flies will not come”* [KTP_10_38F]

**3.3 Subtheme 3: Preventive health check-ups**

Mothers recognized the value of regular health camps and check-ups, describing them as useful for early detection of illnesses such as diabetes, hypertension and anaemia. They reported undergoing blood tests, sputum tests, along with checks for blood pressure, sugar levels, and weight, and were willing to participate whenever such services were available. One mother explained,

*“We are going for health checkups. We go once a month to ‘Belapu’ village government hospital. When camps are arranged, we go there and get our BP and other health checkups done.”* [KTP_10_38F]

**3.4 Subtheme 4: Child hygiene and deworming**

Mothers reported that their children generally followed good hygiene practices at home, including brushing twice daily, trimming nails, washing hands with soap, and wearing footwear outdoors. They stressed that maintaining these habits was essential for protecting children’s health. Additionally, deworming was also considered important, with mothers noting that neglecting it could lead to children becoming thin, developing stomach worms, and suffering from stomach pain, vomiting and fever. One mother explained,

*“After getting up in the morning, she brushes her teeth, washes her face and hands, and then has her tea. In the afternoon she washes her hands and has her lunch. At night she baths, washes her hand and has her food. She also trims her nails.”* [KTP_10_38F]

#### 4. Theme 4: Traditional healing with modern care.

**4.1 Subtheme 1: Intergenerational knowledge and remedies**

Mothers described traditional healing practices as a valuable legacy passed down through generations, particularly from grandmothers. This cultural knowledge, shared orally, has been preserved and actively practiced within households. They reported using a variety of plants for common ailments, such as *‘sambarballi*’ leaves (*Coleus amboinicus*) for headaches, ‘*tumbe*’ leaves (*Leucas aspera*) mixed with onion juice to treat colds, and ‘*sogase*’ leaves (*Adhatoda zeylanica*) for breathlessness. Brahmi (*Bacopa monnieri*) was also believed to enhance memory. As one mother explained,

*“From generations, our grandmothers were following all these, and they have taught us the same. We continue to follow them. [bangles clinging]”* [KTP_10_38F]

Another mother detailed her household practice, *“We use ‘tumbe’ leaves. We add onions to the leaves, grind them, and extract the juice. Once a month, we give this to children. Then there’s ‘sambarballi,’ which we use for headaches. We heat the leaves in the fireplace and place them on our forehead, like this [showing with action]. This reduces our headaches. We also apply the extract on the centre of our head, which helps relieve colds.*” [KTP_14_42F]

**4.2 Subtheme 2: Integration of traditional and modern healthcare practices**

Mothers described a pragmatic approach to managing illnesses, beginning with home remedies for common ailments and turning to doctors if these were ineffective. This reflected a balanced use of traditional wisdom alongside modern medicine in everyday healthcare. As one mother explained,

*“First, we prepare medicines at home. Home remedies. Then, if it doesn’t work, we go to the hospital.”* [KTP_12_34F]

#### 5. Theme 5: Barriers and community solutions.

**5.1 Subtheme 1: Geographic and logistics barrier**

Mothers identified remoteness and lack of transport as major obstacles to accessing healthcare and education. Families living in forested or hilly areas reported difficulties reaching schools and hospitals because vehicles such as autos were unavailable. One mother shared,

*“Aa... [pause] Means how to say, for some people, hospitals are not nearby. Some people’s houses are in remote areas, in forests and on mountains. There they do not get any vehicles like autos to travel. To travel to hospitals also is a problem. If those facilities are given, then it is good.”* [KTP_5_28F]

**5.2 Subtheme 2: Behavioural challenges**

Mothers identified lifestyle factors such as alcohol use, tobacco chewing, poor sleep, and frequent consumption of oily foods as key barriers to good health in their community. These behaviours were seen as harmful habits that risk their well-being. One mother explained,

*“The major challenges are drinking alcohol, chewing gutka and eating oily food.”* [KTP_13_42F]

**5.3 Subtheme 3: Socioeconomic, educational and nutritional barriers**

Some mothers perceived that a lack of education and financial hardship were significant challenges in their community. Schooling opportunities were seen as uneven, with some children able to attend while others dropped out, and many families were unable to afford private schools. Poverty further restricted access to both education and healthcare. Mothers noted that they often lacked the resources to follow doctor’s advice or purchase adequate food. In addition, caste-based discrimination was perceived as an added obstacle that compounded their difficulties. One mother shared,

*“There might be other problems, but we may not know about other people. Education is less. Problems related to education are more. Some people may face these issues, but I don’t know. Some may have less education; education related problem might be there.”* [KTP_12_34F]

Another noted, *“To get proper education, there is poverty, and then there is caste-based discrimination also. These create barriers to education. Then, there is a money problem also.”* [KTP_18_28F]

**5.4 Subtheme 4: Suggested solutions**

Mothers proposed several strategies to overcome barriers to health and education. They emphasized the role of teachers in encouraging families to send children to school. Structural support, such as bus services through ITDP, was seen as essential for improving school access. Mothers also stressed the importance of family and community involvement, noting that stronger guidance could help overcome bad habits such as chewing gutka, using tobacco, and drinking alcohol. One mother stated,

*“The government should do something, so that children don’t drink alcohol and arrange for some rehabilitation centres. At home, elders should not influence their children to drink alcohol. Instead, they should encourage their children to go to school.”* [KTP_16_38F]

## 4. Discussion

This study, the first qualitative exploration of maternal perceptions in the Koraga tribal community of Southern Karnataka, provides insights into how cultural beliefs, government schemes, and structural barriers intersect to shape child health and nutrition. Mothers perceived improvements in their community’s health status, largely attributing this to the role of community health workers such as ASHAs, who provided home visits, raised awareness of healthy lifestyle practices, and linked families to maternal and child benefit schemes. This is in line with the findings by Kalne et al. who reported that community health workers play a pivotal role in enhancing the health outcomes among socially disadvantaged communities in India [26]. At the same time, despite reporting better access to healthcare through ASHAs and valuing government programs such as the *Thayi card* and ITDP, mothers continued to express concerns about child weakness, poor dietary diversity, and behavioural risk factors. This highlights a gap where perceived improvements do not always translate into actual health outcomes, a pattern consistent with findings from other tribal groups in India [13,27].

The study findings can be interpreted through the UNICEF conceptual framework by mapping the identified themes across its causal levels. Basic causes are reflected in “Barriers and community solutions” (Theme 5), which highlight structural constraints such as poverty, caste-based marginalization, and geographic isolation. Underlying causes are captured in “Nutrition beliefs and practices” (Theme 2) and “Hygiene and health promotion” (Theme 3), representing household-level factors including dietary behaviours, hygiene practices and engagement with government schemes. Immediate causes are evident in “Evolving perception of health and wellbeing” (Theme 1) and “Traditional healing with modern care” (Theme 4), which influence child-level outcomes through feeding practices, illness management, and care-seeking behaviours. Together, this mapping demonstrates how maternal perceptions operate across interconnected structural, household, and individual levels to shape child health and nutritional outcomes.

Within this broader structural context, early marriage may also represent a potential underlying determinant in similar marginalized settings. Although not directly assessed in this study, it is well documented that early marriage is associated with reduced maternal education, limited autonomy, and poorer maternal and child health outcomes, including undernutrition and adverse health-seeking behaviours [28,29].

At the household level, mothers’ dietary beliefs further illustrated how these influences shaped everyday practices. Their beliefs regarding foods perceived to increase body heat as well as oily foods, resembled taboos reported in different tribal communities in Karnataka and Odisha [30,31]. While these can prevent overconsumption of unhealthy foods, they may also limit dietary diversity without adequate nutrition education. Additionally, mothers were aware of food safety measures and health risks associated with the consumption of street foods and improperly stored food. This is consistent with the findings of Rao et al., who reported similar mindfulness among rural mothers in other regions of India [32].

Extending beyond diet, mothers placed strong emphasis on hygiene as a core determinant of health. Handwashing, nail trimming, and household cleaning were strongly viewed as influential in shaping children’s habits. Mothers also acknowledged the importance of school-based deworming programs in improving children’s health and increasing parental awareness. This aligns with studies showing that teachers who administer deworming at schools serve as important influencers, indirectly influencing parental health awareness.

Alongside nutrition and hygiene, traditional healing practices reflected the continuity of intergenerational knowledge and their coexistence with modern care. Mothers described using locally available plants for treating common ailments, while also seeking modern healthcare. Koraga mothers combined these remedies with modern care when home remedies were ineffective. This pragmatic approach reflects their trust in both systems. This is consistent with findings from other indigenous groups globally [33]. This duality offers a potential bridge for culturally sensitive interventions, where health workers respect traditional practices while promoting evidence-based care.

Taken together, these findings have several policy implications. While schemes like POSHAN Abhiyan, Integrated Child Development Scheme (ICDS), and Janani Suraksha Yojana aim to improve maternal and child health [34,35], their effectiveness for Koraga families depends on cultural tailoring and delivery adaptations. For instance, ITDP food rations could be diversified to include fresh produce and ASHAs and community elders can be leveraged as cultural mediators, helping bridge modern health care recommendations with local beliefs. Addressing behavioural risks such as alcohol and tobacco use requires integration of family-centred counselling within ITDP and panchayat-level activities.

## 5. Strengths and limitations

The strength of the study lies in its qualitative ethnographic design, which enabled deep engagement with participants and facilitated a thorough, in-depth exploration of their perspectives and cultural context. The study’s credibility was further enhanced by the use of a validated semi-structured interview guide, prolonged immersion in the community and systematic thematic analysis.

Despite its notable strengths, the study also had certain limitations. The reliance on self-reported data from mothers could have introduced bias, as they might have provided responses that they perceived as socially acceptable rather than conveying their actual views. However, efforts were made to minimize this risk through prolonged engagement in the community and rapport-building with participants. This encouraged mothers to speak openly about both positive practices and sensitive issues. Additionally, the results of the study are more pertinent to the Koraga community which makes it difficult to generalize to other tribal communities of India, though they offer valuable transferability to contexts with similar sociocultural and structural conditions.

Furthermore, as the study focused on children aged 5–10 years, direct comparability with national datasets such as the NFHS, which primarily assess children under five years, was limited. However, this focus enabled exploration of an understudied age group and extended understanding of nutritional vulnerabilities beyond early childhood.

## 6. Conclusion

The study highlights the significant influence of maternal perceptions, shaped by traditional beliefs, cultural practices and interactions with community health workers on health and child nutrition among Koraga tribal population. While mothers valued government schemes, safe dietary practices and hygiene, persistent concerns such as child weakness, limited dietary diversity, poverty, and behavioural risks revealed a gap between awareness and actual health outcomes.

The findings underscore the need for context-specific interventions that are both culturally appropriate and operationally feasible. Integrating locally practiced dietary behaviours such as avoidance of “heaty” foods, reliance on home-prepared traditional meals and awareness of food safety along with commonly used home-based remedies for minor illnesses into ASHA-led counselling modules can improve acceptance of recommended nutrition, hygiene, and health-seeking practices. In addition, women’s self-help groups within existing ITDP structures can be used to deliver peer-led nutrition education. These groups can also support behaviour change interventions, particularly to improve dietary diversity and address behavioural risks such as alcohol and tobacco use. School-based platforms, including deworming programs, can further support family-level awareness on hygiene and nutrition. Importantly, these strategies are feasible within the current policy landscape, as they build on existing systems such as ASHA networks, ITDP, and ICDS, requiring contextual adaptation rather than additional infrastructure.

Future research should use longitudinal and mixed-method approaches to examine how maternal perceptions evolve with sustained interventions and their impact on child nutrition.

## Supporting information

S1 File Consolidated criteria for reporting qualitative studies (COREQ): 32-item checklist. (DOC)

S2 File Interview guide. (DOCX)

S3 Table Table 1. Demographic characteristics of study participants (N = 20). (DOCX)

S4 Table Table 2. Illustrative participant quotations from Koraga mothers supporting key themes (N = 20). (DOCX)

S5 File Inclusivity in global research questionnaire. (DOCX)
