# Supplementary material for: Exploring perceptions towards health and child nutrition: A qualitative study among tribal mothers in Southern Karnataka
Source: PLoS One. 2026 Jun 23;21(6):e0351319. doi: 10.1371/journal.pone.0351319 (PMC13289867; doi:10.1371/journal.pone.0351319)
Supplement: S2 File — (DOCX) [file pone.0351319.s002.docx]

Interview Guide

# Title: Interview guide for Assessment of the General Perception on Health and Nutritional Status of Children among Mothers of Koraga Tribal community - A Qualitative Approach

Dear study participant,

Thank you for agreeing to be interviewed.

This research aims to understand the general perception regarding the tribal health and nutrition status of children in the Koraga Tribal community. You are requested to participate in this interview for approximately 30-45 minutes to assess your opinion regarding tribal health, nutritional status, food habits, lifestyle practices, culture related aspects, awareness towards malnutrition and government schemes and policies. You will be asked to respond to the questions asked by the researcher during the session and advise that doing so is voluntary. The interview will be audio-recorded by a research member with your consent.

An informed consent form along with the participant information sheet is provided that describes the research process, confidentiality, and the interview session in detail. This means that your interview responses will only be shared with research team members, and we will ensure that any information regarding identity will be kept confidential.

On behalf of our research team, I would like to extend our gratitude for your time and help. Are there any questions about what I have just explained?

Are you willing to participate in this interview?

Interviewee: Date:

IEC No:

Site:

Start time: End time:

# Demographic details

| 1. | How old are you? | Years |
| --- | --- | --- |
| 2. | How many members are there in your family? | _ |
| 3. | What is your highest education level? | No formal education Primary education Higher secondary Graduation |
| 4. | What is your occupation? | Homemaker Agricultural labour  Government/ Private employee Others |
| 5. | What kind of family structure do you have? | Nuclear Joint Extended |
| 6. | What are the dietary habits of your family? | Veg Mixed |
| 7. | What kind of latrine facility do you have? | Indoor Outdoor |

**Interview questions**

1. Describe what is health? Probes:
   - What factors or aspects of life do you consider when evaluating your own health?
   - Are there any specific factors you believe strongly influence a person's health, and if so, what are they?
   - Are there any cultural or societal beliefs that have shaped your understanding of health?
   - Are there any challenges that people of your Koraga community face in striving to achieve the kind of health you described?
2. Describe the ways of remaining healthy. Probes:
   - Can you share some specific daily habits or practices that you believe contribute to good health?
   - In your opinion, how do lifestyle choices, such as diet and exercise, influence overall health and well-being?
   - What role does preventive healthcare, such as regular check-ups and vaccinations, play in your strategy for remaining healthy?
   - What steps do you take to stay up to date with your health check-ups and screenings.
3. What are the steps taken by you to remain healthy? Probes:
   - Can you share your personal experience with adopting and maintaining a healthy lifestyle?
   - What led you to take these steps?
   - What dietary choices do you find most effective for your own health, and how do you ensure a balanced diet?
   - Are there any stress management techniques or mindfulness practices you use to maintain your mental health?
4. Describe the food items required to remain healthy. Probes:
   - Can you provide specific examples of fruits and vegetables that you believe are essential for a healthy diet?
   - In your opinion, how important are whole grains in maintaining good health, and can you name some specific examples?
   - What specific risks or concerns are associated with allowing cooked food to sit out at room temperature for extended periods?
5. Describe food or food groups that you think people should limit or avoid for the sake of their health?

Probes:

- - What are the specific health concerns or reasons that lead you to recommend limiting or avoiding these foods or food groups?
  - Can you provide examples of healthier alternatives to these foods for people looking to make dietary improvements?
  - Why do you think it's generally recommended to avoid eating food from roadside vendors or street stalls?

1. What are your routine hygienic practices? Probes:
   - In your opinion, how does proper hygiene contribute to overall health and well-being?
   - Can you discuss the importance of hand hygiene and how it can help prevent the spread of illnesses?
   - What role does environmental hygiene, such as cleanliness in your living space, play in your overall hygiene routine?
   - Can you describe any specific health risks or diseases that are associated with open defecation?
   - Are there any cultural or regional aspects that influence your approach to hygiene practices?
2. Could you share specific health concerns you have for your child? Probe:
   - What factors contribute to these concerns?
   - Are there lifestyle factors, such as diet or physical activity, that you believe contribute to your child's health concerns?
3. Could you provide examples of food items that are essential for a child's growth and development?

Probe:

- - What nutrients or benefits do these specific foods offer to children?

1. How do you ensure a balanced diet for your child, including the inclusion of fruits, vegetables, proteins, and grains?

Probe:

- - Describe different types of foods do you typically include in your child's diet
  - Are there strategies or meal plans that you follow to make sure they receive a variety of nutrients?

1. Are there foods or food groups that you believe children should limit or avoid for the sake of their health?

Probe:

- - What specific concerns or risks do you associate with those foods or food groups?
  - In your opinion, what is the potential impact of excessive sugar consumption on a child's health and well-being?

1. How do you handle challenges related to picky eating or dietary preferences with your child?

Probe:

- - Have you found any effective strategies for encouraging a diverse and healthy diet for your child?

1. What role does your cultural or family background play in shaping your child's dietary choices and food traditions?

Probe:

- - Do you incorporate cultural dishes or family recipes into your child's diet? If yes, could you please describe them?

1. Can you describe your child's daily routine when it comes to personal hygiene, such as washing hands or taking baths?

Probe:

- - How do you ensure that these practices become a consistent part of their routine?
  - How do you teach and reinforce the habit of handwashing with children, and what strategies have you found effective?
  - Why is ensuring access to adequate handwashing facilities and hygiene education in schools and public spaces crucial for children's health?

1. In your view, how important is education and awareness in promoting deworming as a routine healthcare practice for children?

Probe:

- - Should parents and caregivers be knowledgeable about the advantages of deworming? If so, what are the reasons behind this belief?
  - What are the potential long-term consequences of neglecting deworming for children's health and development?

1. How do you believe community-based initiatives or government programs can effectively combat open defecation among children?

Probe:

- - Are there successful strategies or campaigns that you've observed in this regard?

1. Do you think malnutrition has affected your child’s growth and overall academic development, especially his/her practical learning? If yes, can you explain in what way?
2. Do you have pets at home? If yes, Probe:
   - Can you discuss how your family members, especially children, are involved in pet hygiene practices and pet care education?
   - How does involving children in pet care help teach them about responsibility and hygiene?
   - In your view, how does pet care impact the overall cleanliness of your home, and how do you maintain a clean environment?
   - In your opinion, how does maintaining good pet hygiene affect children's health and well-being?
   - Are there specific health benefits or risks associated with the level of pet hygiene?
3. Could you share specific examples of traditional healing practices that are commonly used in tribal communities?

Probe:

- - How have these practices evolved over time and adapted to modern healthcare needs?
  - Are there rituals, remedies, or ceremonies that are specially designed for treating ailments in children?

1. Could you provide details about the maternal and child nutritional benefit schemes that you are aware of?

Probe:

- - How did you come to learn about these schemes?
  - Are they widely recognized and easily available to those who require them?
  - What do these schemes typically offer or aim to achieve in terms of maternal and child health?
  - Are there awareness campaigns or community efforts that help inform people about these schemes?

1. How, in your view, does education influence a person's nutritional choices and habits? Probe:
   - How might formal education, like school curricula, influence the development of nutritional knowledge and behaviors in individuals?
   - What challenges or barriers can hinder the influence of education on nutritional choices in your community, and how they might be addressed?
2. According to you what are the main barriers to achieving good health status in your community?

Probe:

- - In your opinion, can education play a role in enhancing one's current health status? Are you willing to adopt good lifestyle practices if introduced to you?
  - Are you familiar with strategies for enhancing health and well-being? If yes, could you share some of the strategies you're aware of?

1. I want to express my gratitude for your time and insights today. Is there anything else you'd like to share from your personal experience regarding practical aspects of your community’s health status before we conclude the interview?
