# Supplementary material for: Exploring perceptions towards health and child nutrition: A qualitative study among tribal mothers in Southern Karnataka
Source: PLoS One. 2026 Jun 23;21(6):e0351319. doi: 10.1371/journal.pone.0351319 (PMC13289867; doi:10.1371/journal.pone.0351319)
Supplement: S3 Table — (DOCX) [file pone.0351319.s003.docx]

**Table 1. Demographics characteristics of study participants (N=20)**

| **S/N** | **Participant ID** | **Age (years)** | **Education level** | **Occupation** | **Family type** | **Eating habits** | **Toilet facility** |
| --- | --- | --- | --- | --- | --- | --- | --- |
| 1 | KTP1 | 42 | Primary | Govt/pvt employee | Joint | Mixed | Indoor |
| 2 | KTP2 | 37 | Primary | Home maker | Nuclear | Mixed | Indoor |
| 3 | KTP3 | 34 | Primary | Home maker | Nuclear | Mixed | Indoor |
| 4 | KTP4 | 29 | Primary | Govt/pvt employee | Joint | Mixed | Indoor |
| 5 | KTP5 | 28 | Higher secondary | Govt/pvt employee | Joint | Mixed | Indoor |
| 6 | KTP6 | 41 | Higher secondary | Home maker | Nuclear | Mixed | Indoor |
| 7 | KTP7 | 37 | Primary | Home maker | Extended | Mixed | Indoor |
| 8 | KTP8 | 35 | Higher secondary | Others | Joint | Mixed | Indoor |
| 9 | KTP9 | 32 | Primary | Home maker | Extended | Mixed | Indoor |
| 10 | KTP10 | 38 | Primary | Others | Extended | Mixed | Indoor |
| 11 | KTP11 | 31 | Primary | Home maker | Nuclear | Mixed | Indoor |
| 12 | KTP12 | 34 | Primary | Home maker | Nuclear | Mixed | Indoor |
| 13 | KTP13 | 42 | Higher secondary | Home maker | Nuclear | Mixed | Indoor |
| 14 | KTP14 | 42 | Higher secondary | Home maker | Extended | Mixed | Indoor |
| 15 | KTP15 | 28 | Higher secondary | Govt/pvt employee | Extended | Mixed | Indoor |
| 16 | KTP16 | 38 | Higher secondary | Govt/pvt employee | Nuclear | Mixed | Indoor |
| 17 | KTP17 | 29 | Primary | Home maker | Nuclear | Mixed | Indoor |
| 18 | KTP18 | 28 | Higher secondary | Home maker | Nuclear | Mixed | Indoor |
| 19 | KTP19 | 36 | Higher secondary | Others | Nuclear | Mixed | Indoor |
| 20 | KTP20 | 29 | Primary education | Home maker | Nuclear | Mixed | Indoor |

*KTP: Koraga Tribe Participant; Govt/Pvt: Government/Private employee*
