# Supplementary material for: Exploring perceptions towards health and child nutrition: A qualitative study among tribal mothers in Southern Karnataka
Source: PLoS One. 2026 Jun 23;21(6):e0351319. doi: 10.1371/journal.pone.0351319 (PMC13289867; doi:10.1371/journal.pone.0351319)
Supplement: S4 Table — (DOCX) [file pone.0351319.s004.docx]

| **Table 2. Illustrative participant quotations from Koraga mothers supporting key themes (N=20)** | | | |
| --- | --- | --- | --- |
|  | | | |
|  | | | |
| **S/N** | **Themes** | **Subthemes** | **Illustrative Quotations** |
|  | Evolving perception of health and wellbeing | Improved access and health status | *“Now it is fine, there is no difficulty in health like before. Now, the healthcare centre gets to know about it, it is good for people who have BP and sugar. They come to the house, because of which it is better now.”* [KTP_19_36F]  *“Now presently it is good. If any problems come, we advise them not to do that way. Among us only we keep advising each other don’t eat unhealthy food, don’t eat food that causes allergies. We all follow it. So, there is not many problems.”* [KTP_10_38F] |
|  |  | Holistic wellbeing practices | *Describe the ways of remaining healthy.?* [I]  *“We should eat vegetables, fish and meat. Then, we should walk. We must have our food on time. Sleep. We should sleep well and drink plenty of water at regular intervals. That’s all.”* [KTP_11_31F]  *I: Are you familiar with strategies for enhancing health and well-being?*  *KTP: yes.* [KTP_13_ 42F]  *I: if yes, can you tell which and all?*  *KTP: We should eat good food, it is good if we exercise, then meditation. We should not eat oily food.* [KTP_13_ 42F] |
|  |  | Societal influence on health beliefs | *“when we go for medicines, doctors say that we should wash our hands using soap, we should take bath, nails should be clean, should wear chappals while going out. [ladies talking], should not play in mud. Even in schools’ teachers tell this. ASHA workers come home and tell us all this.”* [KTP_11_ 31F]  *“Nurses come home, and doctors too visit us. We observe, and they too tell us.”* [KTP_13_ 42F] |
|  | Nutrition beliefs and practices | Safe and balanced diet | *“Green gram, eggs and all we should give. Milk should be given to children after they finish their meal and before they sleep. They eat bananas also. That’s also good for health know. Then, nonveg food items like chicken and fish we should give.”* [KTP_1_42F]  *“We should not eat more oily food; Stomach worm will develop. Should eat vegetables, leafy vegetables, green gram, eggs, fish, meat, and pomegranate.”* [KTP_11_31F] |
|  |  | Cultural food traditions | *“We make ‘gatti’ using different leaves like banana leaves and turmeric leaves. Then, we prepare ‘pathrode’, ‘dosa’ made up of ‘kesu’ and drumstick leaves and give it to our children; that’s also good.”* [KTP_1_42F]  *“Traditional food [birds chirping] like Dosa we prepare. Idli, ‘pundi’, ‘gatti’. Banana leaf ‘gatti’ then jackfruit ‘gatti’ also I prepare. Banana leaf ‘gattis’ are prepared more.”* [KTP_13_42F] |
|  |  | Feeding strategies for children | *I: what measures or steps do you take to ensure that your child is getting all different types of nutrients?*  *So that they eat everything. Now you told pomegranate and all right that they must eat now if they don’t eat it how will you make sure that they get the nutrition of pomegranate?*  *if they don’t eat it, I will make juice of it in mixer by adding some milk.* [KTP_1_42F] |
|  |  | Government nutrition support | “*We get food, you know-jaggery, and all. For our Koraga community we get monthly nutritious food from -ITDP-. In that, we get chickpeas, toor dal, jaggery, eggs and ghee.”* [KTP_14_42F]  *“We are getting food from -ITDP- also. So, it has benefitted us in that way”.* [KTP_6_42F] |
|  | Hygiene and health promotion | Government schemes and role of education | *“Could you provide details about the maternal and child nutritional benefit schemes that you are aware of?”* [I]  *“[birds chirping] Thayi card. It has been very useful for us. We have been following that.”* [KTP_13_42F]  *“According to you how important is education in reducing stomach worm in children?”* [I]  *“Yes, education is important. It is very important. If stomach worm increases, children’s stomach starts to pain, they will vomit. All this we understand and get them medicines.”* [KTP_10_38F] |
|  |  | Household and environmental hygiene | *“For dust, it is good to clean. We should not allow dirty water to stay for long time; otherwise, mosquitoes will increase, and they will bite us and cause problems for our health. So, I fear that. If we keep clean, then flies will not come”* [KTP_10_38F]  *“If environment is clean then mosquitoes will be less, and flies will not come. Like that if environment is clean then I feel it is good. Then what else. That’s all.”* [KTP_12_34F] |
|  |  | Preventive health check-ups | *“We are going for health checkups. We go once a month to ‘Belapu’ village government hospital. When camps are arranged, we go there and get our BP and other health checkups done.”* [KTP_10_38F]  *“Doing medical tests when they do check-up, they check everything here in government hospital. People from govt hospital come here and do blood check-up, check up and ask about our full health.”* [KTP_3_34F] |
|  |  | Child hygiene and deworming | *“After getting up in morning, she brushes her teeth, washes her face and hands, and then has her tea. In the afternoon she washes her hands and has her lunch. At night she baths, washes her hand and has her food. She also trims her nails.”* [KTP_10_38F]  *“Morning after waking up he will brush his teeth, then wash his face, has his food and sometimes he takes bath and goes to school. He cuts his nail once in a week. That’s all. After using toilet, he will wash his hands using soap. [child talking].”* [KTP_12_34F] |
|  | Traditional healing with modern care | Intergenerational knowledge and remedies | *“In the past, our elders used to teach us remedies. Grandmothers told us that if children have a headache, heating 'sambarballi' leaves and placing them on the forehead helps. They also suggested putting ground carom seeds on the centre of the head.”* [KTP_15_28F] |
|  |  | Integration of traditional and modern healthcare practices | *“First, we prepare medicines at home. Home remedies. Then, if it doesn’t work, we go to the hospital.”* [KTP_12_34F]  “*For cold and fever if we give extract of ‘tumbe’ leaves for some people it will reduce. For many people it has reduced. We believe that even for our children it will reduce. Even with this if it doesn’t reduce then we go to doctor.”* [KTP_10_38F] |
|  | Barriers and community solutions | Geographic and logistics barrier | *“Education and hospital. we don’t have hospitals nearby. and it is far for people to go, it is difficult.”* [KTP_6_41F]  *“hmm.. schools are not nearby, and hospitals are also not nearby. Vehicles don’t come. Roads are not proper.”* [KTP_7_37F] |
|  |  | Behavioural challenges | *“Some people drink alcohol, chew tobacco, tambaku. From these habits they spoil their health.”* [KTP_12_34F] |
|  |  | Socioeconomic, educational and nutritional barriers | *“Difficulty means we see some children getting sick and staying at home. Some parents don’t send their children to schools. There are problems. Related to money. They can’t afford sending their children to private schools.”* [KTP_13_42F]  *“To get proper education, there is poverty, and then there is caste-based discrimination also. These create barriers to education. Then, there is money problem also.”* [KTP_18_28F] |
|  |  | Suggested solutions | *“In our community if they don’t drink alcohol, we are much healthy. My wish is that everybody should get education. if we have education, we will know everything. We will have confidence to go everywhere. That is my experience. Some people drink alcohol, chew tobacco that is how their health gets spoilt. If all these reduces, we will be healthy I feel. That’s all.”* [KTP_12_34F]  *“Families and community organizations should provide support, but sometimes people don't attend meetings. If families offer support, many issues can be resolved. In these meetings, the topic of sending children to school is frequently raised.”* [KTP_14_42F] |

*I: Interviewer; KTP: Koraga tribe participant; Subscript (e.g., 18): participant number; Age (e.g., 28): participant age in years; F: gender of participant (F for females) so, KTP_18_28F represents ‘Koraga tribe participant 18, aged 28, female’.*
